# Life cycle of phytoreoviruses visualized by electron microscopy and tomography

**DOI:** 10.3389/fmicb.2013.00306

**Published:** 2013-10-16

**Authors:** Naoyuki Miyazaki, Atsushi Nakagawa, Kenji Iwasaki

**Affiliations:** ^1^Institute for Protein Research, Osaka UniversityOsaka, Japan; ^2^National Institute for Physiological SciencesOkazaki, Japan

**Keywords:** electron microscopy, electron tomography, virus structure, *Rice dwarf virus*, *Rice gall dwarf virus*

## Abstract

*Rice dwarf virus* and *Rice gall dwarf virus*, members of the genus *Phytoreovirus *in the family *Reoviridae*,**are known as agents of rice disease, because their spread results in substantial economic damage in many Asian countries. These viruses are transmitted via insect vectors, and they multiply both in the plants and in the insect vectors. Structural information about the viruses and their interactions with cellular components in the life cycle are essential for understanding viral infection and replication mechanisms. The life cycle of the viruses involves various cellular events such as cell entry, synthesis of viral genome and proteins, assembly of viral components, viral egress from infected cells, and intra- and intercellular transports. This review focuses on the major events underlying the life cycle of phytoreoviruses, which has been visualized by various electron microscopy (EM) imaging techniques, including cryo-electron microscopy and tomography, and demonstrates the advantage of the advanced EM imaging techniques to investigate the viral infection and replication mechanisms.

## INTRODUCTION

The life cycle of viruses involves various cellular events including cell entry, synthesis of viral genome and proteins, assembly of viral components, and viral egress from infected cells. Structural information about viruses and their interactions with cellular components are essential for understanding viral infection and replication mechanisms. Transmission electron microscopes are commonly used to obtain two-dimensional (2D) projection images from thin sections of resin-embedded virus-infected cells, which enable visualization of viral and virus-related structures, as well as the virus localization within host cells. Thus, conventional electron microscopy (EM) has long been used to provide valuable insights into virus–host interactions and the life cycle of viruses inside host cells. However, projection causes overlap in the features, resulting in the intrinsic ambiguities in the interpretation of the 2D projection images of three-dimensional (3D) objects. Electron tomography (ET) is used to generate 3D structures from 2D EM images projected in various directions. Thus, the overlaps that appear in the 2D projection images can be resolved in the reconstructed 3D structures, thereby enabling effective and precise analysis of interactions between a virus and host cell. In this review, we describe the life cycle of phytoreoviruses as revealed by various EM imaging techniques, including cryo-electron microscopy (cryo-EM) and ET, and immunofluorescence microscopy and immunoelectron microscopy. Phytoreoviruses were chosen for this review because they are the most widely studied of all plant viruses that are currently threatening the stable production of cereal crops (Omura and Mertens, 2005).

*Rice dwarf virus* (RDV), *Rice gall dwarf virus *(RGDV), and *Wound tumor virus* are members of the genus *Phytoreovirus* in the family *Reoviridae*. These viruses are icosahedral double-shelled particles with an average diameter of approximately 70 nm (Takahashi et al., 1994; Miyazaki et al., 2005), and they are known as serious agents of rice diseases that cause economic damage in many Asian countries. Phytoreoviruses are transmitted to rice plants via insect vectors, and they multiply both in the plants and in the invertebrate insect vectors. RDV is the most well-characterized virus among the three phytoreoviruses. The capsid structure of RDV was previously determined at a resolution of 3.5 Å using X-ray crystallography (Nakagawa et al., 2003), and the structural organization of the capsid shell was characterized by cryo-electron single-particle analysis (Miyazaki et al., 2010). RDV has a 12-segmented, double-stranded RNA (dsRNA) genome encoding 12 viral proteins (**Table 1**), and the RDV virion is composed of seven structural proteins (P1, P2, P3, P5, P7, P8, and P9). P3 forms the inner capsid shell, which encloses the viral genome, and P1, P5, and P7 are involved in transcription (Hagiwara et al., 2003, 2004; Nakagawa et al., 2003; Miyazaki et al., 2010). The inner capsid shell is surrounded by the outer capsid shell, which is composed of P2, P8, and P9 (Omura et al., 1989; Omura and Yan, 1999; Zhong et al., 2003). Five non-structural proteins (Pns4, Pns6, Pns10, Pns11, and Pns12) are associated with the replication cycle of RDV within host cells, which involves synthesis of the viral genome and proteins, assembly of progeny viruses, and intercellular movement, among other processes. Three non-structural proteins Pns6, Pns11, and Pns12 are the constituents of viroplasms. Viroplasms are the viral inclusion bodies appearing in the cytoplasm of RDV-infected cells, which is believed to be the primary site of virus replication and assembly (Wei et al., 2006c). RDV Pns4 is a phosphoprotein localized around the viroplasms and is known to form bundles of minitubules at later stages of infection; however, its function remains to be clarified (Wei et al., 2006b). RDV Pns10 forms tubular structures containing virus particles, and it is directly involved in the intercellular spread of RDV among insect vector cells. These observations have shown that the life cycle of RDV is tightly controlled by these non-structural proteins, which facilitates efficient virus proliferation in host cells.

**Table 1:** Viral proteins of *Rice dwarf virus*.

Protein	Molecular weight (kDa)	Location	Function
P1	164	Structural, inside of the capsid shell	RNA polymerase
P2	127	Structural, outer capsid	Vector transmissibility
P3	114	Structural, inner capsid	Core capsid
Pns4	83	Non-structural	Unknown
P5	91	Structural, inside of the capsid shell	Guanylyltransferase
Pns6	56	Non-structural	Nucleic acids binding, viroplasm matrix protein in insect vector cells, cell-to-cell movement in plants
P7	55	Structural, inside of the capsid shell	RNA-binding
P8	46	Structural, outer capsid	Outer capsid
P9	39, 30	Structural, outer capsid	Unknown
Pns10	35	Non-structural	RNA silencing suppressor in plants, intercellular transport in insect vector cells
Pns11	23	Non-structural	Viroplasm matrix protein, nucleic acids binding
Pns12	34	Non-structural	Viroplasm matrix protein

## VIRUS ENTRY

Initiation of a successful viral infection and replication cycle requires viral attachment to specific molecules on the surface of host cells, with subsequent entry into the host cells for delivering the viral genome and proteins required for replication. The steps involved in virus entry into host cells have recently become the most widely studied aspect of the life cycle of animal viruses, including human pathogenic viruses. Animal viruses exploit various endocytosis pathways, including clathrin-mediated endocytosis, caveola-mediated endocytosis, macropinocytosis, and phagocytosis, to enter the host cell cytoplasm. On the other hand, plant viruses are believed to enter plant cells through a wound on plants, although the cell entry pathways of plant viruses remain unclear owing to the lack of a suitable experimental system.

*Rice dwarf virus* is transmitted to plants by vector insects, primarily the green rice leafhopper, in a persistent-propagative manner. The vector insect acquires the virus by feeding on virus-infected plants. After ingestion by leafhoppers, the viruses first accumulate in the epithelial cells of the filter chamber in the alimentary canal of the leafhoppers, which suggests that the microvillar membrane of the filter chamber might contain abundant cellular receptors for viral attachment and entry (Chen et al., 2011). RDV proliferates in the vector insect, and the insect becomes RDV-infected after a latent period of approximately 2 weeks. Subsequently, RDV is transmitted to plants by the viruliferous vectors, most likely via a wound caused by feeding of the insects on the plants. The minor outer capsid protein P2 is essential for RDV infection to the vector insects (Yan et al., 1996; Tomaru et al., 1997; Omura et al., 1998). Viral particles containing P2 on the outer capsid layers can infect insect vectors through direct injection and by feeding insects through a membrane, whereas viral particles without P2 can infect insect vectors only through direct injection. Furthermore, non-sense mutations in the P2 gene inhibit transmission of the virus from infected plants to insect vectors (Pu et al., 2011), and the P2 protein induces membrane fusion in insect host cells (Zhou et al., 2007). These evidences suggest that P2 attaches to undefined receptors on insect vector cells and mediates viral cell entry (Omura et al., 1998).

**FIGURE 1:**
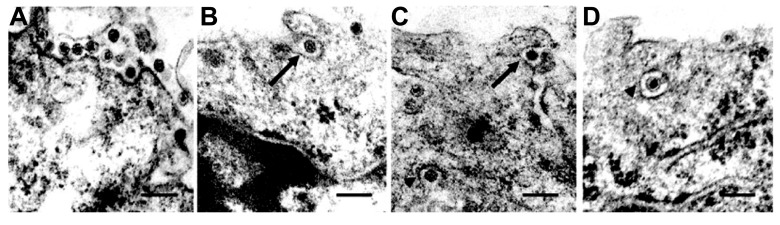
**Cell entry of *Rice dwarf virus* (RDV) via the clathrin-mediated endocytosis pathway.** **(A)** Attachment of RDV particles to the plasma membranes of vector cell monolayers (VCMs) 30 min p.i. **(B,C)** Uptake of RDV particles in coated pits (arrows) 1 h p.i. **(C,D)** RDV particles internalized within coated pits (arrowheads) 1 h p.i. Reproduced with permission from Wei et al. (2007). Scale bars, 200 nm.

Established monolayer cultures of leafhopper vector cells (NC24 cells), originally derived from embryonic fragments dissected from eggs of *Nephotettix cincticeps*, enable further investigation of the results of earlier studies on RDV infection and the cytopathology of infected cells. The vector cells in monolayers (VCMs) were inoculated with RDV, fixed at different post-inoculation (p.i.) times, and then examined by EM to analyze the cell entry pathway. These investigations revealed that RDV entry into the insect vector cells occurred via a clathrin-mediated endocytosis pathway (**Figure 1**; Wei et al., 2007). The cell entry process via coated pits was further confirmed by the significant reduction in RDV infectivity after the cells were treated with drugs that block receptor-mediated or clathrin-mediated endocytosis (Wei et al., 2007). The EM investigations also showed that RDV within endocytotic vesicles remained as intact double-layered particles. This process differs from that of animal reoviruses such as rotavirus, bluetongue virus, and mammalian reovirus, in which cell entry involves a series of molecular transformations in the outermost layer of proteins, which strips these proteins from the virion and delivers the inner capsid particle in a transcriptionally active form into the cytosol.

## VIRUS REPLICATION AND ASSEMBLY

**FIGURE 2:**
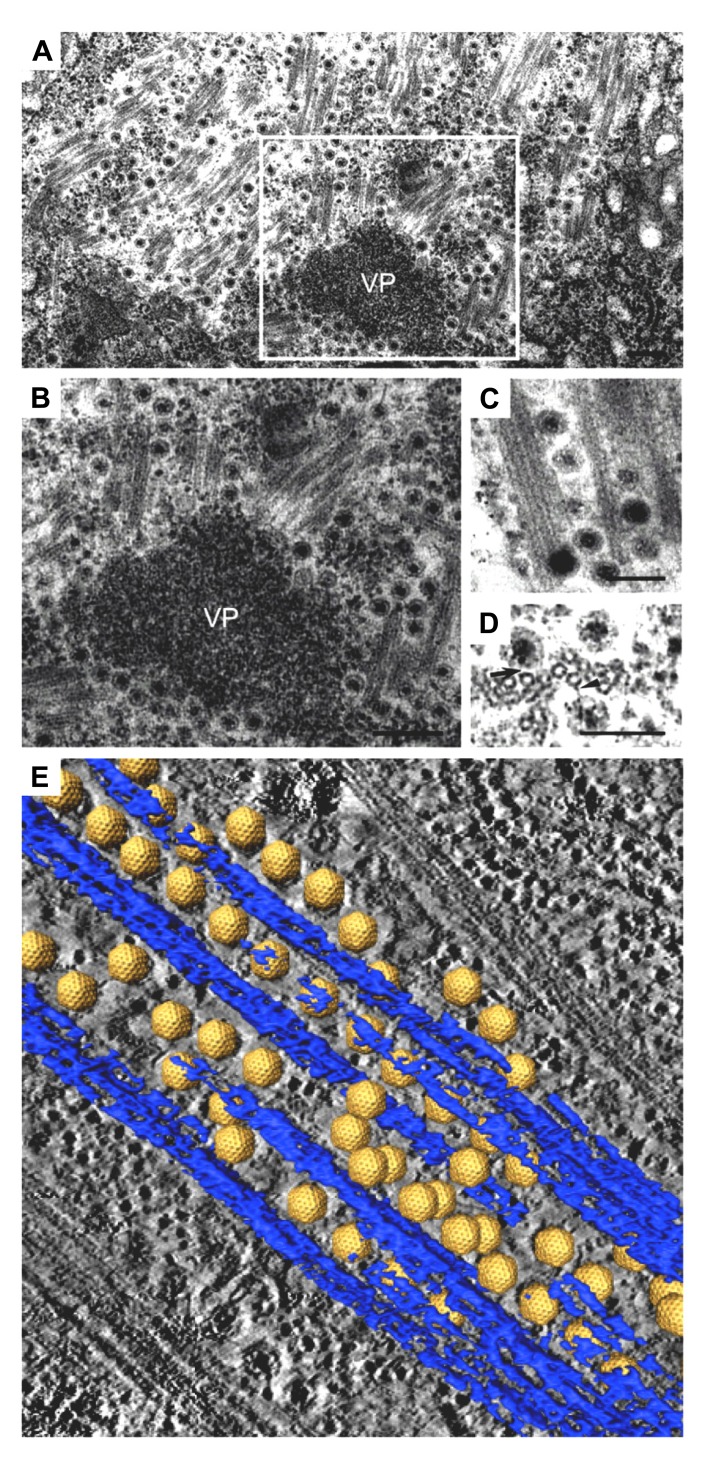
**Intracellular trafficking of *Rice gall dwarf virus* (RGDV) particles.** **(A)** RGDV particles associated with microtubules in virus-infected VCMs 48 h p.i. Scale bar, 300 nm. Viroplasm (VP), an electron-dense inclusion, is also seen in the electron micrograph. **(B)** Enlarged view of the boxed region in **(A)**. Scale bar, 300 nm. **(C)** Viral particles along the edges of tubules, approximately 25 nm in diameter. Scale bar, 150 nm. **(D)** Transverse sections of arrays of closed circles of approximately 25 nm in diameter with viral particles directly attached to their circumference (arrow) or via a filament (arrowhead). Scale bar, 150 nm. **(E)** Three-dimensional surface-rendered model showing the association of RGDV particles (yellow) with microtubules (blue). Reproduced with permission from Wei et al. (2009b).

After cell entry, viruses initiate their replication processes. Cytoplasmic viral inclusions, known as viroplasms, viral factories, or viral inclusion bodies, commonly appear as electron-dense structures in the cytoplasm of cells infected by members of the family *Reoviridae*, and these are therefore assumed to be the site of virus replication (**Figures 2A,B**). RDV viroplasms are formed by three major constituents: the non-structural proteins Pns6, Pns11, and Pns12 (Wei et al., 2006c). Pns12 is essential for the formation of viroplasms; it was shown to possess an intrinsic ability to form aggregates (viroplasm-like structures) when expressed in the absence of any other viral proteins in *Spodoptera frugiperda* (Sf9) cells (non-hosts of RDV). On the other hand, Pns6 and Pns11 are distributed diffusely throughout the cytoplasm, and neither Pns6 nor Pns11 forms aggregates when expressed alone in Sf9 cells. Furthermore, transgenic rice plants expressing proteins that interfere with Pns12 expression were found to be strongly resistant to RDV infection (Shimizu et al., 2009). These observations strongly suggested that viroplasm matrix proteins and viroplasm formation play important roles in viral replication and morphogenesis (Isogai et al., 1998).

To date, two crystal structures (NSP2 of rotavirus in the genus *Rotavirus* and P9-1 of *Rice black streaked dwarf virus* (RBSDV) of the genus *Fijivirus*) and one cryo-EM structure (Pns9 in the genus *Phytoreovirus*) have been identified in the viroplasm matrix proteins of viruses in the family *Reoviridae* (Jayaram et al., 2002; Akita et al., 2011, 2012). The RGDV Pns9 is homologous to RDV Pns12, with only 16% identity between amino acid sequences. These matrix proteins form similar octameric structures, despite the lack of significant similarities between the respective primary and secondary structures or between domain foldings. The similarities of octamer and large aggregate formations among reoviruses imply that the structure of each octamer and its interior pore is crucial for the proper functioning of respective proteins in the viroplasm, for example, in viral morphogenesis. Furthermore, octamerization of RBSDV P9-1 is required for the formation of the matrix of viral inclusions in the cell (Akita et al., 2012). The rotavirus non-structural protein NSP2 is the best-characterized matrix protein in the family *Reoviridae*, and it forms viroplasms in the presence of another non-structural rotavirus protein, NSP5 (Fabbretti et al., 1999; Eichwald et al., 2004). Several biochemical properties of NSP2 have been identified, including RNA-binding, helix- destabilizing, nucleotide triphosphatase, 5′-RNA triphosphatase, nucleoside diphosphate kinase, and core protein-binding activities. Structural analysis by cryo-EM has further revealed that these activities are performed inside a cleft between the two domains of NSP2 and that NSP5 regulates the binding of NSP2 to RNA (Jiang et al., 2006). These findings suggest that NSP2 contributes to packaging and replication of the viral genome by relaxing the secondary structures that impede polymerase function and by facilitating the translocation of viral RNAs into progeny core particles (Jiang et al., 2006).

In addition to Pns12 of RDV, Pns6 and Pns11 are constituents of viroplasms, although neither protein has aggregate-forming ability. Pns6 has an RNA-binding motif, and although it is involved in intercellular movement at plasmodesmata in plants, its function in viroplasms remains unclear. Pns6 binds preferentially to single-stranded RNAs derived from the consensus 5′- and 3′-terminal sequences of the RNA genome as described below, and it is considered to form ribonucleoprotein complexes to transport the viral genome between plant cells. Pns11 is also a nucleic acid-binding protein (Xu et al., 1998), and newly synthesized viral RNAs accumulate within viroplasms. These RNA-binding activities and colocalization within the viroplasm suggest that Pns6 and/or Pns11 might be recruited to viral inclusions through their association with Pns12, thereby indicating that Pns6 and Pns11 might play an important role in viral RNA transport to the viroplasms or in viral RNA synthesis and replication in the viroplasms (Akita et al., 2012).

Virion components are also accumulated and sorted in and around the viroplasms, suitable for the assembly of progeny viral particles. Components for the viral core particles (P1, P3, P5, and P7 proteins) are located in the interior region of viroplasms, and progeny core particles have been observed within the viroplasms. In contrast to genomic RNAs and core component proteins, accumulation of the outer capsid proteins P2, P8, and P9 is evident in the peripheral regions of the viroplasms, and intact double-layered viral particles are commonly observed around the viroplasms, which are considered newly synthesized progeny viruses. These observations suggest that core particles are constructed inside the inclusions, whereas outer capsid proteins are assembled at the periphery of the inclusions. This hierarchical assembly model of the viral particle coincides with results by structural analysis of the capsid proteins at atomic resolutions and by biochemical experiments (Nakagawa et al., 2003; Hagiwara et al., 2004; Miyazaki et al., 2005).

## INTRACELLULAR TRANSPORT OF VIRIONS

After multiplication of the viruses at the replication sites, progeny viruses move to the periphery of cells to be released from viroplasms. Various animal viruses have been shown to utilize cytoskeletal motor proteins for their intracellular movements (reviewed in Sodeik, 2000; Döhner et al., 2005; Greber and Way, 2006; Radtke et al., 2006). Motor proteins mediate intracellular transport along cytoskeletal filaments (actin fibers and microtubules). Microfilament motor proteins such as myosin move along the filaments through interactions with actin molecules, whereas microtubule motor proteins such as dynein and kinesin move along the microtubules through interactions with α- and β-tubulin molecules. Dynein complexes are much larger and more complex than kinesin and myosin, and the complexes move to the minus-end of the microtubules (retrograde). Kinesins typically contain two heavy chains with motor heads that move along microtubules either toward the plus-end or toward the minus-end, depending on the specific type of kinesin involved.

The association of RGDV particles with microtubules can be seen clearly in EM images, and the 3D structures can be analyzed with ET (**Figure 2**; Wei et al., 2009b). Although RGDV particles are aligned on microtubules, they do not directly attach to the microtubules. Therefore, a gap between viral particles and microtubules is present, and a rod-like structure can be seen in the gap, which is considered a motor protein for the transportation of viral particles. Furthermore, depolymerization of microtubules using the pharmacological drugs nocodazole and colchicine resulted in decreasing the number of viruses released from the infected cells to 1/5th that released from untreated cells without significantly reducing the production of cell-associated viruses. These results suggested that the microtubule motor protein is involved in the transport of viral particles from their replication site to the cell surface for the viral egress from the infected cell, but it is not involved in transport from the cell surface to the replication site during cell entry. Based on its direction of movement to the cell surface, the rod-like structure appears to be a plus-end-directed (anterograde) kinesin motor protein. Kinesin-1 is the only anterograde microtubule motor known to be involved in the intracellular transport of viruses to date (Rietdorf et al., 2001; Jouvenet et al., 2004; Greber and Way, 2006). The size of the connecting density between RGDV particles and microtubules also supports the involvement of kinesin rather than the relatively bulky dynein complex (Iwasaki and Omura, 2010). This cytosolic transport system is similar to those of some animal viruses. For example, in the case of murine polyomavirus, EM observations revealed associations of viral particles with the free end and the lateral sides of microtubules (Sanjuan et al., 2003), which indicated that intracellular transport was mediated by the interaction between viral particles and microtubules. This was confirmed by the observation that microtubule depolymerization prevented polyomavirus migration both toward the nucleus and from the nucleus to the cell surface.

## INTERCELLULAR MOVEMENT IN INSECT VECTORS

Phytoreoviruses multiply both in plants and in vector insects, but utilize different strategies for spreading in the two hosts. In insects, the non-structural protein Pns10 is involved in the intercellular movement of RDV. Pns10 forms tubular structures enclosing viral particles. RDV containing tubular structures in association with the microvilli of the midgut in viruliferous vector insects are frequently observed in EM analysis (Nasu, 1965; Chen et al., 2012). An established cell culture system and immunolabeling for viral gene products enabled further detailed analysis of the intercellular movement of RDV that exploits the unique tubular structure. In the cultured insect cells, Pns10 tubules extended from the infected cell and colocalized with actin filaments in the filopodia. Direct interaction of Pns10 with actin molecules was detected by surface plasmon resonance, and actin-depolymerizing drugs suppressed the extension of Pns10 tubules from the cell surface (Wei et al., 2006a). These results suggested that viral spread into neighboring cells occurred by direct cell-to-cell contact via filopodia, without diffusion through the extracellular environment, as evidenced in animal viruses such as African swine fever virus (Jouvenet et al., 2006) and murine leukemia virus (Lehmann et al., 2005; Sattentau, 2008). These results were further confirmed using virus-neutralizing antibodies. When virus-neutralizing antibodies were added to the medium, they captured cell-free viruses and strongly inhibited virus spreading through the extracellular environment (Wei et al., 2006a). However, RDV could spread directly from the initially infected cell to adjacent cells, even in the presence of virus-neutralizing antibodies.

**FIGURE 3:**
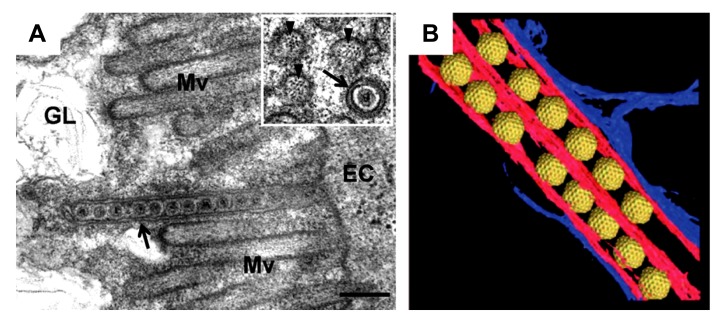
**Intercellular transport of *Rice dwarf virus* (RDV) in insect vector cells exploiting virus-induced Pns10 tubular structures.** **(A)** Virus-containing Pns10 tubules associated with microvilli of anterior midgut in viruliferous leafhoppers. Inset, transverse section of microvillus containing a virus-packed Pns10 tubule approximately 100 nm in diameter. EC, epithelial cell; GL, gut lumen; Mv, microvilli. Scale bar, 200 nm. **(B)** Three-dimensional surface-rendered model showing RDV particles (yellow) surrounded by Pns10 tubules (red) and the plasma membrane (blue). Reproduced with permission from Katayama et al. (2007) and Chen et al. (2012).

The 3D structure of the association between Pns10 tubules and RDV particles in the filopodia was determined by ET (Katayama et al., 2007; **Figure 3**). The inner diameter of the Pns10 tubule corresponds very well with the maximum diameter of the RDV particle (75 nm), and the virus particles are tightly packaged inside the Pns10 tubule. The tightly packed virus particles did not appear to diffuse inside the Pns10 tubule freely. Furthermore, the tip of the short tubules protruding from the surface was always filled with virus particles. If RDV particles are loaded inside Pns10 tubules after formation of the tubular structures, empty Pns10 tubules should be observed, and packed viral particles should be more scattered toward the tip of the tubules. These observations suggest that tubule extension might be mechanically linked with virus loading. After formation of the virus-containing tubule structure, it becomes associated with the actin filaments and is then transferred to neighboring cells through interactions with actin molecules in the filopodia. When the virus-containing tubule structure surrounded by cellular membrane reaches the neighboring cells, membrane fusion between the two cells is required for the intercellular transport of RDV. Although the mechanism has been unknown, but P2 protein of RDV may be needed for membrane fusion for RDV to enter the neighboring cells, because the P2 protein is a membrane fusion protein (Zhou et al., 2007).

## INTERCELLULAR MOVEMENT IN PLANTS

The intercellular movement of viruses in plants is completely different from that in animals; plant viruses move to adjacent cells via plasmodesmata. The plasmodesma is an intercellular junction unique to plants, and it directly connects the cytoplasms of adjacent cells that are separated by a rigid and thick cell wall. Plasmodesmata allow the passage of molecules with molecular mass less than ~800 Da and mediate intercellular communication across the thick cell wall. Plant viruses exploit this intercellular communication pathway to enable their own spread within the infected plant. In order to facilitate the cell-to-cell movement through plasmodesmata, many plant viruses encode specific movement proteins that can modify the plasmodesmatal size-exclusion limit (Wolf et al., 1989) or the plasmodesmatal structure, e.g., tubule formation inside the plasmodesma (Laporte et al., 2003). Virion or viral ribonucleoprotein complexes can be transported through the modified plasmodesmata into adjacent cells. In the case of RDV in vector insects, Pns10 was found to be involved in the intercellular movement between neighboring insect cells through formation of tubular structures, as described above (Wei et al., 2006a). On the other hand, in plants, Pns10 acts as an RNA silencing suppressor (Cao et al., 2005; Ren et al., 2010; Zhou et al., 2010), and it is not essential for multiplication (Pu et al., 2011). Instead, Pns6 is primarily responsible for the movement of RDV between plant cells (Li et al., 2004; Ji et al., 2011).

Pns6 preferentially binds to single-stranded RNAs derived from the consensus 5′- and 3′-terminal sequences of the RDV genome and to non-specific dsRNA. The sense-strand RNAs from all genome segments of RDV contain the conserved 5′- and 3′-terminal sequences, 5′-GGGAAA–- or 5′-GGUAAA–- and –-UGAU-3′ or –-CGAU-3′, respectively (Kudo et al., 1991). Many other reoviruses have similar specific consensus sequences at the ends of each segment of their genomic dsRNAs, which implies functional significance and their possible biological roles, although these remain to be clarified. Pns6 is localized to the plasmodesmata, whereas the RDV virion is not found in the plasmodesmata. In addition, since the size of the RDV virion is much larger than the pore size of plasmodesmata, RDV is considered to move between plant cells in the form of viral ribonucleoprotein complexes containing Pns6 proteins. Moreover, Pns6 has ATPase activity, similar to other movement proteins found in numerous plant viruses (Peremyslov et al., 1999). The ATPase activity of the movement proteins is thought to be necessary to provide the driving force to traffic viral RNA through plasmodesmata or to suppress RNA silencing (Howard et al., 2004; Bayne et al., 2005). However, the molecular mechanism underlying the function of intercellular movement proteins remains unclear, and it should be investigated in future studies using EM imaging tools.

## EGRESS

Viruses must be released from infected cells for their successful spreading. In the case of RDV, multiple pathways have been reported for viral egress from infected insect cells without cell lysis. One of the virus-release pathways involves secretory exosomes derived from multi-vesicular bodies (MVBs; Wei et al., 2008b, 2009a). Virus-containing MVBs are frequently observed in the peripheral region of the viroplasms as well as near the cytoplasmic membrane. Analyses using organelle-specific markers showed that the virus-containing vesicles are late-endosomes or lysosomes. Furthermore, actin filaments and myosin motors have been shown to affect the morphology and motility of virus-containing MVBs in infected cells (Wei et al., 2008a). Based on these results, the following RDV release pathway was proposed. Newly synthesized viral particles, which assemble at the periphery of the viroplasm, are engulfed by MVBs and moved along the cytoskeletal actin filaments to the periphery of cells by myosin motors. At the periphery, these vesicular bodies fuse with the plasma membrane in an exocytic manner to release viral particles out of the cell, thereby enabling spreading. However, further analysis is required to confirm this pathway. Virus spreading from infected insect vector cells through the virus-release pathway via exosomes and the intercellular transporting pathway using Pns10 tubular structures causes less damage to the host insect cells than virus spreading involving cell lysis does (e.g., egress of many other non-enveloped viruses). Therefore, RDV appears to use the host cell machinery to replicate itself, but it does not interfere with the vitality of the host organism, thus ensuring the survival of the vector insect. The survival of the vector insect increases the opportunity for RDV to be transmitted to plants, which widens the scope of viral spread and increases viral survival.

## APPLICATIONS OF CRYO-ELECTRON TOMOGRAPHY FOR VISUALIZING VIRAL INFECTION AND REPLICATION MECHANISMS

**FIGURE 4:**
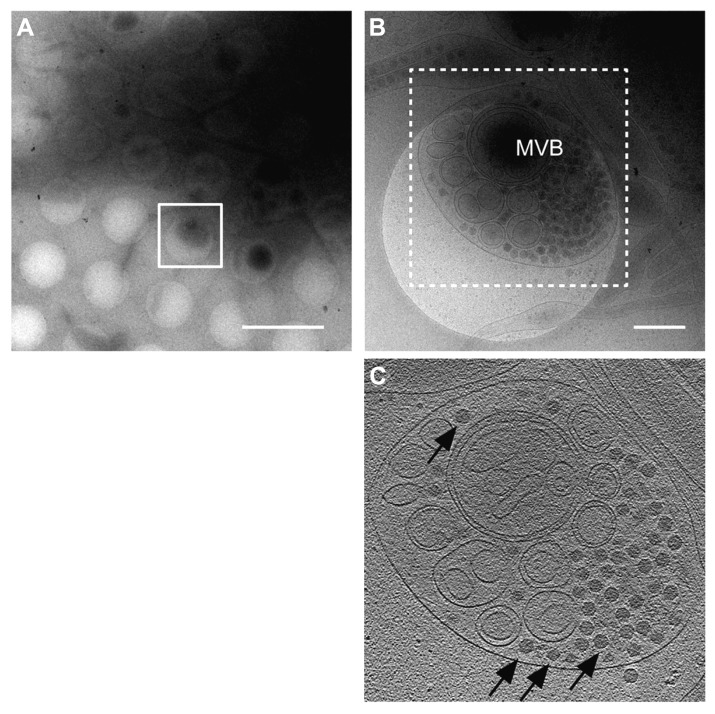
**Virus egress from infected insect cells.** Cryo-electron microscopy (cryo-EM) images of a RDV-infected insect cell cultured on the EM grid. **(A)** Low magnification cryo-EM image of the RDV-infected insect cell cultured on the EM grid. Scale bar, 2 μm. **(B)** High magnification cryo-EM image of the boxed region in **(A)**. Scale bar, 400 nm. **(C)** A slice through the reconstructed tomographic volume obtained from the area highlighted in **(B)**. Black arrows indicate some of the RDV particles within the multi-vesicular body (MVB). Reproduced with permission from Miyazaki et al. (2013).

To understand the life cycle of a virus, it is necessary to conduct a detailed investigation of the viral and virus-related structures and to analyze their molecular interactions within a cellular context. cryo-EM and cryo-electron tomography (cryo-ET) can be used to visualize fully hydrated cells in a close-to-native state at molecular resolutions, which allows analysis of molecular interactions within a cell in more detail than that using conventional EM and ET (Robinson et al., 2007). Recently, cryo-EM/ET has been applied to visualize some animal viruses and bacteriophages within their host cells. For example, morphological changes of the vaccinia virus before and after intrusion into the host cells (Cyrklaff et al., 2007) and cell entry and intracellular trafficking of the herpes simplex virus were captured by cryo-EM/ET (Maurer et al., 2008; Ibiricu et al., 2011). We also attempted to apply the cryo-EM/ET method in the RDV study (Miyazaki et al., 2013). NC24 cells were cultivated and infected with RDV on the EM grid, frozen in liquid ethane, and then embedded in vitreous ice. When the cells were examined by cryo-EM, RDV particles within multi-vesicular compartments at the edge of the cell were clearly visible (**Figure 4**). These results demonstrate the success of approaches using cryo-EM/ET techniques for observing the viral and virus-related structures within cells and for analyzing the viral life cycle around the peripheries of infected cells. In the near future, advanced cryo-EM/ET methods will be applied to not only animal viruses, but also to various plant viruses, which will extend our understanding of fundamental biological processes of viruses.

## CONCLUSION

**FIGURE 5:**
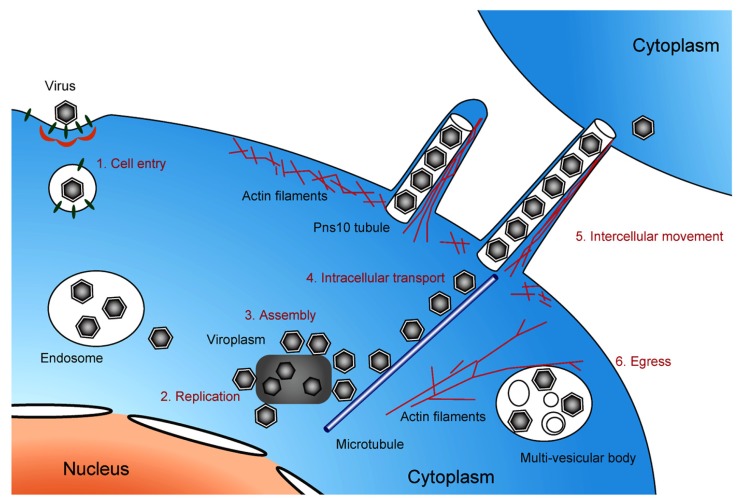
**Life cycle of pytoreoviruses in the host cell.** A schematic illustration showing the life cycle of phytoreoviruses in the host insect cell, involving viral cell entry via clathrin-mediated endocytosis (1), replication of viral proteins and genome (2), assembly of progeny viruses in and around viroplasm inclusions (3), microtubule-associated intracellular transport (4), intercellular movement exploiting Pns10 tubule (5), and viral egress via multi-vesicular bodies (6).

Electron microscopy imaging techniques, including the cryo-ET approach described herein, have been used for high-resolution analysis of viral and virus-related structures within cells. This review describes the major cellular events occurring in the life cycle of phytoreoviruses, the main agents of rice diseases, which were primarily elucidated through EM imaging (**Figure 5**). However, EM observations could be enhanced with other complementary methods that allow observations of dynamic events, such as confocal laser scanning microscopy, which would provide more information. Furthermore, applications of the advanced cryo-EM/ET technology to virus research have enabled more detailed investigations of viral infection and replication mechanisms. We believe that in the near future, further use of these approaches will reveal the molecular mechanisms underlying infection and replication of viruses that are currently threatening the stable production of cereal crops and will further extend our understanding of these viruses.

## Conflict of Interest Statement

The authors declare that the research was conducted in the absence of any commercial or financial relationships that could be construed as a potential conflict of interest.
